# The Stature of Boys Is Inversely Correlated to the Levels of Their Sertoli Cell Hormones: Do the Testes Restrain the Maturation of Boys?

**DOI:** 10.1371/journal.pone.0020533

**Published:** 2011-06-02

**Authors:** Kirstie Morgan, Nicola A. Dennis, Ted Ruffman, David K. Bilkey, Ian S. McLennan

**Affiliations:** 1 Department of Anatomy & Structural Biology, Brain Health Research Centre, University of Otago, Dunedin, New Zealand; 2 Department of Psychology, University of Otago, Dunedin, New Zealand; University of Muenster, Germany

## Abstract

The testes of preadolescent boys appear to be dormant, as they produce only trace levels of testosterone [1]. However, they release supra-adult levels of Müllerian Inhibiting Substance (MIS, anti-Müllerian hormone) and lesser levels of inhibin B (InhB), for unknown reasons [2], [3]. Boys have a variable rate of maturation, which on average is slower than girls. The height of children relative to their parents is an index of their maturity [4], [5]. We report here that a boy's level of MIS and InhB is stable over time and negatively correlates with his height and his height relative to his parent's height. This suggests that boy's with high levels of MIS and InhB are short because they are immature, rather than because they are destined to be short men. The levels of MIS and InhB in the boys did not correlate with known hormonal modulators of growth, and were additive with age and the growth hormone/IGF1 axis as predictors of a boy's height. If MIS and InhB were causal regulators of maturity, then the inter-boy differences in the levels of these hormone produces variation in maturation equivalent to 18-months of development. MIS and InhB may thus account for most of the variation in the rate of male development. If boys lacked these hormones, then an average 5-year-old boy would be over 5 cm taller than age-matched girls, making boys almost as dimorphic as men, for height. This indicates that boys have a high growth potential that is initially suppressed by their testes. The concept of the childhood testes suppressing an adult male feature appears paradoxical. However, the growth of children requires intergenerational transfer of nutrients. Consequently, the MIS/InhB slowing of male growth may have been historically advantageous, as it would minimizes any sex bias in the maternal cost of early child rearing.

## Introduction

The testis has two endocrine cells: The steroid-producing Leydig cells and the Sertoli cells that secrete Müllerian Inhibiting Substance (MIS, anti-Müllerian hormone) and inhibin B (InhB). The production of these hormones varies during development, causing the hormonal profile of the testes to vary substantially during the major developmental stages.

The testes produce high levels of testosterone during the second semester of gestation, infancy and from puberty [1], [6]. In contrast, preadolescent boys only have trace levels of testosterone, which overlap extensively with the levels of testosterone in girls [1]. This has led to the presumption that the testes of boys are functionally dormant. The Sertoli cells of boys, however, secrete supra-adult levels of MIS [2], [7] and lesser levels of InhB into the blood [3]. Both of these hormones are highly dimorphic during childhood [2], [3].

MIS triggers the degeneration of the uterine precursor in male embryos around the 8^th^ week of gestation [8], but high production of MIS continues until puberty [2], [7]. MIS is thus the most abundant testicular hormone during childhood, for both humans and other mammals. The function of childhood MIS is largely obscure, although recent studies with *Mis^−/−^* mice suggest that MIS may contribute to the subtle sex biases in the brain and behaviour [9], [10], [11]. InhB is part of the gonadal-pituitary axis that regulates sperm production in men. Boys do not produce sperm, but their Sertoli cells nevertheless produce InhB, with the plasma levels of InhB in boys being typically less than half that of men [3].

Embryos are initially asexual, with the male phenotype emerging under the influence of testicular hormones. We therefore hypothesize that the Sertoli cell hormones induce a male bias in developmental processes that occur during childhood. Boys mature more slowly than girls, with the average duration of boyhood (time between infancy and the pubertal growth spurt) being about two years longer than the duration of girlhood [4]. We report here that boys with high levels of MIS and/or InhB are short relative to the height of their parents, suggesting that Sertoli cell hormones slow the maturation of boys.

## Results

**Figure 1:**
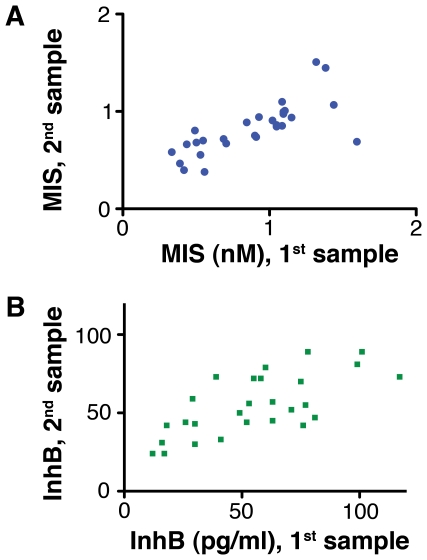
The levels of MIS (A) and InhB (B) were relatively invariant between blood samples taken from boys one year apart. The correlation between the first and second blood samples were MIS, R = 0.74, p<0.0005, n = 28 (see also Lee et al (4)) and InhB, R = 0.69, p<0.0005, n = 27.

The plasma levels of InhB and MIS in 5- and 6-year-old boys were highly variable amongst boys (Fig. S1, S2), but the levels of the hormones in a given boy varied little between two samples collected one year apart (Fig. 1, see also [2]). This constancy suggests that Sertoli cell hormones may regulate processes that are both protracted and relatively constant, such as maturation.

### Childhood height negatively correlates with Sertoli cell hormone

**Figure 2:**
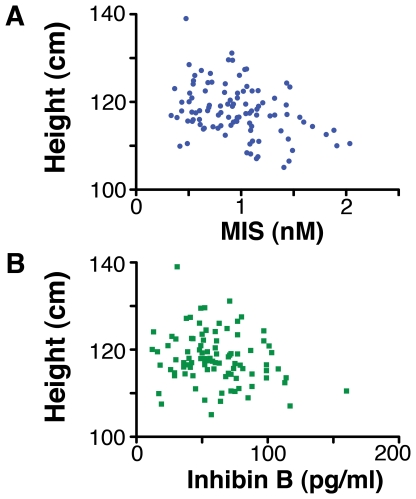
The level of MIS (A) and InhB (B) in 5- and 6-year-old boys negatively correlated with their height. The linear correlation between the hormones and height were height (cm) = 122.9−5.5 * MIS (nM), R = −0.34, p<0.0005, n = 103; and height (cm) = 121.1−0.05 * InhB (pg/ml) R = −0.29, p = 0.004, n = 103.

If InhB or MIS retard aspects of maturation, then traits that increase with age, such as height, should negatively correlate with a boy's levels of hormones. Consistent with this notion, the heights of the boys were negatively correlated with their levels of MIS (Fig. 2a, R = −0.34, p<0.0005) and with their levels of InhB (Fig. 2b, R = −0.29, p = 0.004). Regression analysis showed a slight decrease in both hormones with age, which was statistically significant for MIS (Fig. S1). The effect of age was, however, small (18% per year for MIS) compared to the intra-individual variation of the hormones (range: 0.33–2.04 nM, a 6-fold difference). Hence, the association between height and the Sertoli cell hormones remained strong when corrected for the confounding effect of age, using partial correlation (InhB, R = −0.25, p = 0.013; MIS, R = −0.24, p = 0.016). The BMI (weight/height^2^) and Benn index (weight/height) were not correlated with either of the Sertoli cells hormones, indicating that InhB and MIS are associated with height rather than mass or body composition (Table S1).

**Figure 3:**
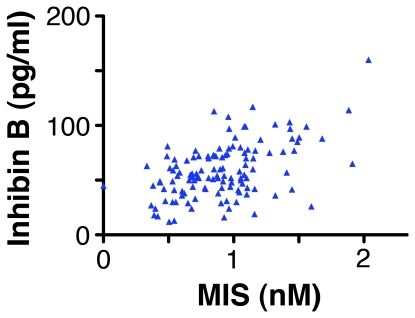
The levels of MIS in the blood of boys are correlated with their level of InhB. The levels of the hormones were correlated with each other, R = 0.48, p<0.0005, n = 96.

InhB and MIS are both secretions of Sertoli cells and a boy's levels of these hormones were correlated (Fig. 3, R = 0.48, p<0.0005). However, this correlation accounts for less than 25% of the variation in the boy's levels of hormones, which is insufficient to explain why InhB and MIS both correlate with height with similar strength. In linear regression models, InhB and MIS reduced each other's partial correlation with height, but did not entirely extinguish it, suggesting that InhB and MIS are redundant regulators of height. If so, the combined effect of InhB and MIS should relate to height more strongly than either hormone alone. To test this idea statistically we normalised each hormone to its mean concentration (to account for the different Kd to their receptors) and added them. The sum of the normalised hormones correlated to height (R = −0.35, p<0.0005) more strongly than either hormone alone, with the correlate being even stronger when each boy's highest normalised value of the two hormones was used (R = −0.40, p<0.0005). These correlations remained strong when age was corrected for using partial correlation (sum of hormones R = −0.29, p = 0.005; highest value R = −0.32, p = 0.001).

### MIS and InhB may correlate with developmental maturity

The height of boys is subject to two independent forces. Height is strongly genetic in populations with access to good nutrition [12]. Consequently, boys can be short because they have short parents and are destined to be small adults [13]. Alternatively, children who are slow to mature are short for their age, but the final adult height of slow and fast maturing boys are not different on average [4], [14]. We therefore examined the relationship between the boys' InhB and MIS levels and the heights of their parents, as a means of determining their relationship to the abovementioned forces.

**Figure 4:**
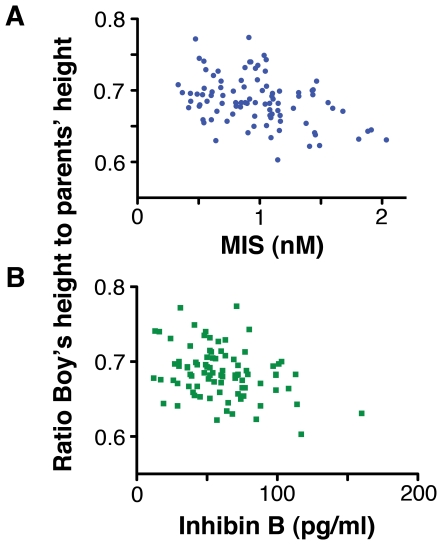
The levels of MIS (A) and InhB (B) in 5- and 6-year-old boys were negatively correlated with their height relative to the average height of their parents. The levels of the boys' hormones were negatively correlated with the value of their height divided by the average height of his parents: MIS, R = −0.35, p = 0.001, n = 96; InhB, R = −0.35, p = 0.001, n = 96. The heights of the boys' parents did not correlate with the boys' levels of MIS (Fig. S5) or InhB (Fig. S4).

The ratio of a boy's height to that of his parents is a measure of his maturity. This ratio had a strong positive correlation with the age of the boys (R = 0.621, p<0.0005), as would be expected given that age is a prime driver of maturity. Conversely, MIS and InhB were negatively correlated to the ratio (Fig. 4a,b) with the correlations remaining strong when the potentially confounding effect of age is controlled for, using partial correlation (InhB, R = −0.31, p = 0.004; MIS, R = −0.23, p = 0.025).

The height of boys in this study correlated with that of their parents (R = 0.33, p = 0.001)(Fig. S3), as would be expected. However, in marked contrast to this, there was an almost perfect disassociation between parental height and a boy's levels of Sertoli cell hormones (InhB, R = 0.03, p = 0.776 Fig. S4; MIS R = 0.01, p = 0.910, Fig. S5). This is consistent with a boy's adult height being independent of his childhood levels of MIS and InhB, although a longitudinal study is needed to establish whether adult male height is independent of boyhood levels of MIS and InhB.

### MIS and InhB may be novel regulators

We then examined whether the levels of Sertoli cell hormones in the boys correlated with their growth hormone/IGF1 axis and/or other hormones that can affect growth. The effects of growth hormone on bone are mainly mediated through IGF1, with modulation through various binding proteins. The IGF1/IGFBP3 ratio is used as an index of the strength of the growth hormone/IGF1 pathway [15]. The levels of MIS and InhB in the boys did not correlate with their levels of IGF1, the IGF1/IGFBP3 ratio or other growth-associated hormones (Table S2). Furthermore, InhB and MIS were additive with IGF1 and the IGF1/IGFBP3 ratio as predictors of a boy's height (Table 1). Significantly, the partial correlations of both InhB and MIS in linear regression models were not reduced by the inclusion of IGF1 and thyroid hormones in the models (Table 1).

**Table 1:** Linear regression models for the prediction of the height of a boy.

	Models including MIS				Models including InhB			
Model	Predictor	Model	Part correlate		Predictor	Model	Part correlate	
		R =	R =	p =		R =	R =	p =
1	MIS	0.34	−0.34	0.000	InhB	0.29	−0.29	0.004
2	Age	0.63	0.52	0.000	Age	0.62	0.55	0.000
	MIS		−0.19	0.016	InhB		−0.20	0.013
3	Age	0.74	0.46	0.000	Age	0.74	0.46	0.000
	IGF1		0.44	0.000	IGF1		0.42	0.000
	MIS		−0.15	0.040	InhB		−0.17	0.022
4	Age	0.80	0.45	0.000	Age	0.80	0.43	0.000
	IGF1		0.44	0.000	IGF1		0.42	0.000
	MIS		−0.21	0.006	InhB		−0.20	0.004
	Mat height		0.24	0.002	Mat height		0.26	0.000
	Pat height		0.11		Pat height		0.13	
	iPTH		−0.04		iPTH		−0.02	
	T3		0.05		T3		0.04	
	T4		0.09		T4		0.06	

“Mat. Height” is the height of the mothers, whereas “Pat. Height” is the height of the fathers. Similar results were obtained when IGF1/IGFBP3 replaces IGF1 in the models. When both MIS and InhB were included the correlation coefficients for models 2&3 were 0.63, 0.75 and 0.81, respectively. The number of boys examined is listed in Table S1.

## Discussion

The testes of boys are generally thought to have little or no influence on them during preadolescent childhood. However, in this study the heights of 5- and 6- year-old boys were negatively correlated with the levels of their Sertoli cell hormones, MIS and InhB (Fig. 2). This suggests that the boyhood testes have an active endocrine role that has been overlooked, because the trait(s) regulated are distinct for the classical testosterone-dependent traits that most overtly define the male phenotype.

### Sertoli cell hormones may influence the duration of childhood

There are two distinct mechanisms that govern whether a boy is short or tall. Most obviously, the height of boys is related to their future adult height, but childhood height also reflects how far the child has progressed in his journey to adulthood. Boys who mature early are tall relative to those who mature more slowly, but the speed of maturation has no effect on final height [4], [14].

In this study, boys with higher levels of MIS and InhB appeared to be shorter because they were maturing more slowly. The ratio between a boy's height and his parent's heights is an index of how far he has progressed towards his adult height. The boy's chronological age correlated with this ratio, whereas MIS and InhB were negatively correlated: that is, MIS and InhB had the opposite effect to age, suggesting that they retard maturation. In contrast, the boy's levels of MIS and InhB did not correlate with the height of their parents, which is a measure of their future adult height.

The levels of MIS and InhB varied considerably between boys, which may explain why the rate of maturation of boys is highly variable. If MIS and InhB are causal regulators, then the average effect of any level of MIS and InhB can be calculated from the linear regression line in Fig 2. The difference between the boys with the highest and lowest levels of MIS was 1.7 nM, which is estimated to create a size difference of 9.3 cm, which is equivalent to 18 months of growth.

### Inhibin B and MIS may have redundant actions

InhB and MIS correlated with the height of the boys to a similar extent, even though the levels of InhB and MIS in boys were only partially correlated (Fig. 3). This suggests that InhB and MIS are redundant regulators, particularly as the sum of a boy's levels of InhB and MIS correlated more strongly with his height than either hormone alone. The hallmark actions of InhB and MIS are independent of each other. However, mice that lack both *Mis* and the *inhibin A* subunit develop testicular cancer more rapidly than mice with a null mutation of one of the genes alone [16]. This indicates that MIS and InhB are convergent regulators of some cell types, although the mechanism underlying this convergence is currently unknown.

MIS and InhB are male hormones during development and boys mature more slowly than girls. This is consistent with MIS and InhB being causal regulators of the speed of maturation, but experimental manipulation of MIS and InhB is needed to prove this. A false correlation could arise if a boy's MIS and InhB levels were regulated by the growth hormone/IGF1 axis, or by some other hormonal regulator of growth. However, a boy's level of Sertoli cell hormones did not correlate with his levels of other hormones. Furthermore, the strength of MIS as a predictor of a boy's height was not degraded by inclusion of IGF1 and his parental height in linear regression models. This suggests that a boy's levels of Sertoli cell hormones are not significantly linked to any determinant of his future height. If so, the models used to predict the future height of boys may be improved by the inclusion of the boy's levels of MIS and InhB.

InhB and MIS have been separately used to estimate Sertoli cell number in the testes. However, the boys' levels of InhB and MIS only partially correlated (R = 0.48), indicating that only 25% of the variation in these hormones is from a common source. This suggests that the amount of InhB and/or MIS produced per Sertoli cell is regulated and varies from boy to boy during middle childhood. Alternatively, the release and/or excretion of these hormones may be differentially regulated.

### Boys may have a high intrinsic growth rate

If the observed relationship between Sertoli cells hormones and height is causal, then the average boy in the study would have been 5.2 cm taller on his 5^th^ birthday, if he had lacked InhB and MIS. This would create a boy-girl difference in height of 5.0%, which is only slightly less than the sex difference in the heights of their parents (7.1±5.3%, mean ± SD). This suggests that boys on average are able to grow more rapidly than girls, but that their testes suppress this ability during pre-adolescent childhood. The control of adult size varies from organ to organ and is poorly understood for bone. However, the adult size of some tissues is partially due to the regulation of cell number in utero [17], [18]. It is therefore possible that the bones of boys are primed for growth by the embryonic exposure to testicular hormones. Alternatively, the Y chromosome has been postulated to be growth promoting [19], although the sequencing of the Y chromosome has not lead to the identification of putative growth-promoting gene.

The hallmark functions of both the embryonic and adult testes relate to the induction of the sex differences, including the generation of a male bias in size. It is thus counterintuitive that the testes of boys may prevent the emergence of a male-bias in size during childhood. We therefore make some preliminary comments on why this might be biologically useful.

The evolutionary pressures that govern the height of boys are likely to be multifaceted, and may be centred on the dependency of a child on their parents. Taller boys have a greater mass than shorter boys (Fig. S6), and their development therefore involves increased utilization of energy and nutrients, including calcium for bone mineralization. The burden of providing these nutrients mainly falls on the mother [20], especially in pre-agricultural environments where nutrient supply is limited. Consequently, an average boy could only be taller than an average girl if there was a significant sex-bias in the inter-generational transfer of nutrients. This would be expected to impact on the reproductive success of their mothers [20], [21], [22] and the mineralization of her bones [23]. Similarly, boys with high growth rates risk malnutrition if their growth outstrips the capacity of their parents to support it. In this context, it is noteworthy that the levels of MIS in boys decline as they age [2], with a time course that parallels the increased capacity of children to gather food in contemporary hunter-gatherer societies [24], [25].

In conclusion, the levels of the Sertoli cell hormones in boys are negatively associated with their height, with high statistical certainty. The biological significance of these observations is less certain, and will require further investigation. This notwithstanding, the data challenges the notion that the testes of boys' are dormant.

## Methods

### Boys

The study included 103 boys who were aged either 5- or 6-years old. Four additional boys were excluded from the study because they elected not to donate blood, after their height was measured. The boys were recruited from local schools and by advertisements in local newspapers. The University of Otago's Human Ethics Committee approved the project.

Each boy's height and weight were measured using standard paediatric measures (National Health and Nutrition Examination Manual, 2007). Height was measured to the nearest 0.1 cm using a portable stadiometer (Seca 214) and weight was measured to the nearest 0.1 kg using electronic scales (Seca 770 High Capacity Scale). Caregivers provided additional information including parental heights.

### Hormone assays

Blood samples were collected at the Southern Community Laboratories Ltd (Dunedin, New Zealand), where a registered phlebotomist collected up to 2 ml of blood into BD vacutainers. The blood sat at room temperature for between 30 minutes and 1 hour, after which the serum was removed, divided into aliquots, snap frozen and stored at −80°C until assay. Twenty-eight of the boys donated a second blood sample, 12±1 month after their initial donation. The blood sample collected closest to the boy's 6^th^ birthday was used for the main study.

The levels of hormones were measured using commercial ELISA, according to the manufacturers' instructions. The ELISAs were: MIS, Beckman Coulter, A73819; IGF1, R&D systems, DG100; IGFBP3, R&D systems, DGB300; InhB, Beckman Coulter, A81301; intact PTH(1–84), ALPCO, 21-IPTH-E01; free triiodothyronine, ALPCO, 25-FT3HU-E01, and free thyroxin, ALPCO, 25-FT4HU-E01. The MIS levels were measured for all boys. There was insufficient serum from 7 boys to measure all hormones. The sample size therefore varied between 96 and 103 for some analyses, as documented in the Figure legends and the Results.

Statistical calculations were done with PASWstatistics, and included linear regression, partial correlations, and linear curve estimates.

## Supporting Information

Figure S1 **The level of MIS in the plasma of 5- and 6-year-old boys is plotted against their age.** Each dot is the value for an individual boy (n = 103). The mean value was 0.95 nM (133 ng/ml), the median 0.92 nM, the standard deviation 0.37 nM, with the individual values ranging from 0.33 to 2.04 nM. The relationship between age and MIS was MIS (nM) = 2.02−0.17* Age (yr), R = −0.26, p = 0.007. See also Lee MM et al. (1996) Mullerian inhibiting substance in humans: normal levels from infancy to adulthood. J Clin Endocrinol Metab 81∶571–576; Aksglaede L. et al. (2010) Changes in Anti-Mullerian Hormone (AMH) throughout the life span: A population-based study of 1027 healthy males from birth (cord blood) to the age of 69 years. J Clin Endocrinol Metab 95∶ 5357–5364.
(TIF)

Figure S2 **The level of InhB in the plasma of 5- and 6-year-old boys is plotted against their age.** Each dot is the value for an individual boy (n = 103). The mean value was 63 pg/ml, the median 58 pg/ml, the standard deviation 34 pg/ml, with the individual values ranging from 12–275. The relationship between age and InhB was InhB (pg/ml) = 112.6−8.2 * Age (yr), R = −0.13, p = 0.195. See also Andersson AM, Skakkebaek NE (2001) Serum inhibin B levels during male childhood and puberty. Mol Cell Endocrinol 180∶103–107.
(TIF)

Figure S3 **The height of 5- and 6-year-old boys is plotted against the average height of their parents.** The height of the boys was significantly correlated with that of their parents, R = 0.33, p = 0.001, n = 97.
(TIF)

Figure S4 **The boys'** **level of InhB is plotted against the height of their parents.** The two variables are not significantly correlated, R = 0.03, p = 0.776.
(TIF)

Figure S5 **The boys'** **level of MIS is plotted against the height of their parents.** The two variables are not significantly correlated, R = 0.01, p = 0.910.
(TIF)

Figure S6 **The height of the boys is plotted against their weights.** The height and weight of the boys were significantly correlated, R = 0.83, p<0.0005.
(TIF)

Table S1 **Relationship between Sertoli cell and other hormones and weight, corrected for the boy**'**s age.** The number of boys examined is listed in Table S2.
(DOC)

Table S2 **Relationships between Sertoli cell hormones and other determinants of the heights of boys.** Abbreviations: IGF1, insulin like growth factor 1; IGFBP3, IGF binding protein 3; iPTH, intact parathyroid hormone; T3, triiodothyronine; T4, thyroxine.
(DOC)
