# Supplementary figures and images for: The Stature of Boys Is Inversely Correlated to the Levels of Their Sertoli Cell Hormones: Do the Testes Restrain the Maturation of Boys?
Source: PLoS One. 2011 Jun 2;6(6):e20533. doi: 10.1371/journal.pone.0020533 (PMC3107220; doi:10.1371/journal.pone.0020533)

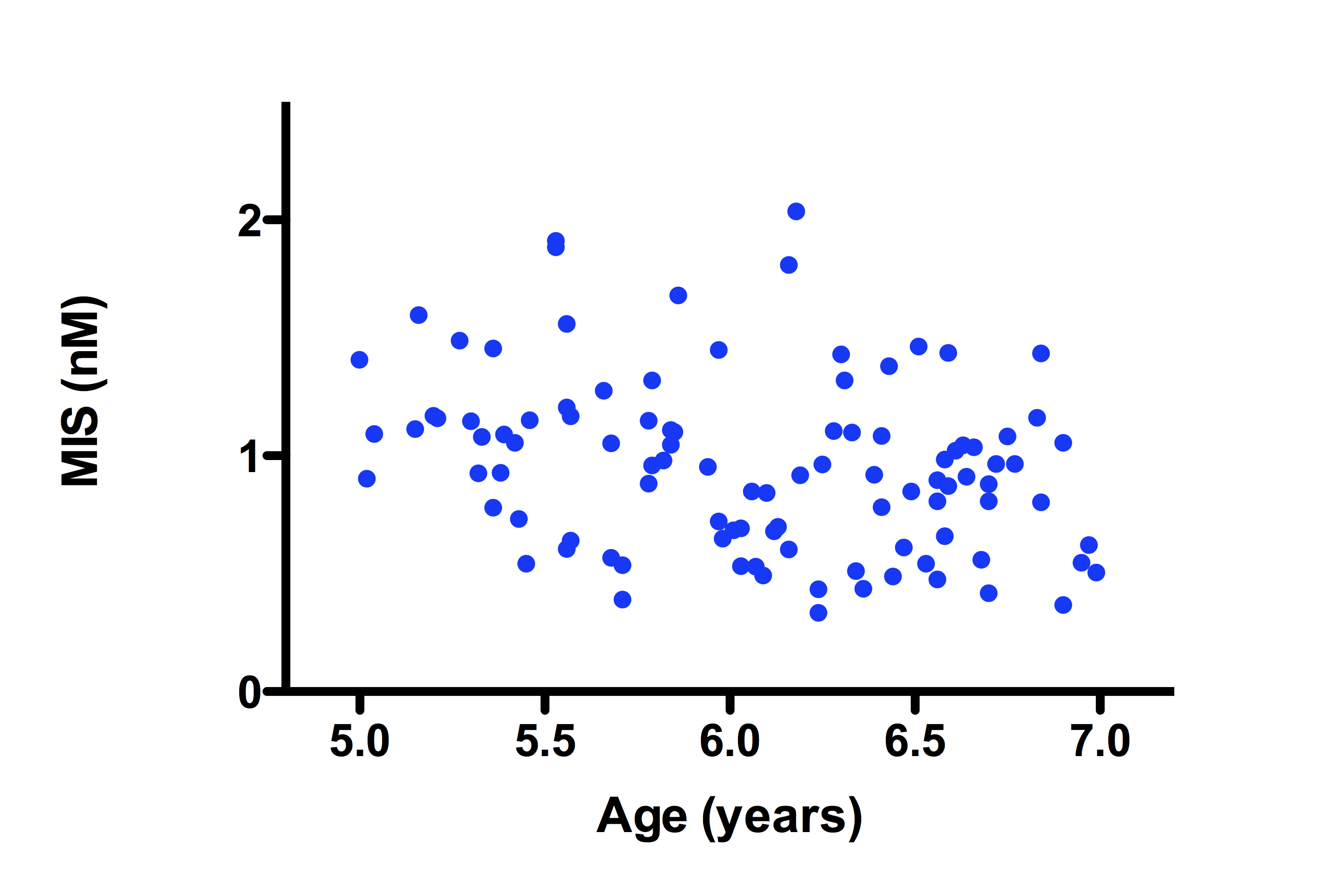

Supplement: Figure S1 — The level of MIS in the plasma of 5- and 6-year-old boys is plotted against their age. Each dot is the value for an individual boy (n = 103). The mean value was 0.95 nM (133 ng/ml), the median 0.92 nM, the standard deviation 0.37 nM, with the individual values ranging from 0.33 to 2.04 nM. The relationship between age and MIS was MIS (nM) = 2.02−0.17* Age (yr), R = −0.26, p = 0.007. See also Lee MM et al. (1996) Mullerian inhibiting substance in humans: normal levels from infancy to adulthood. J Clin Endocrinol Metab 81∶571–576; Aksglaede L. et al. (2010) Changes in Anti-Mullerian Hormone (AMH) throughout the life span: A population-based study of 1027 healthy males from birth (cord blood) to the age of 69 years. J Clin Endocrinol Metab 95∶ 5357–5364. (TIF) [file pone.0020533.s001.tif]

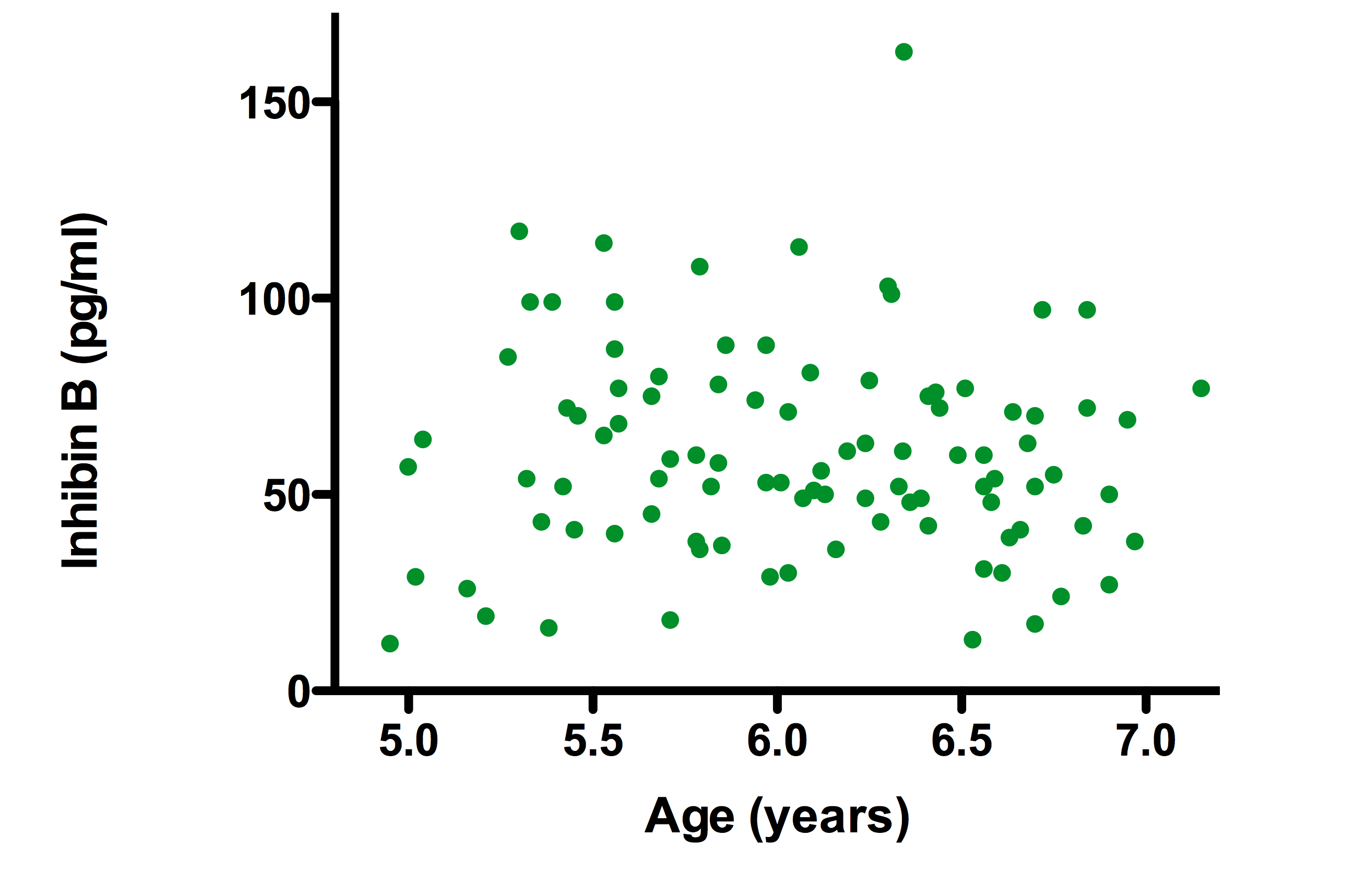

Supplement: Figure S2 — The level of InhB in the plasma of 5- and 6-year-old boys is plotted against their age. Each dot is the value for an individual boy (n = 103). The mean value was 63 pg/ml, the median 58 pg/ml, the standard deviation 34 pg/ml, with the individual values ranging from 12–275. The relationship between age and InhB was InhB (pg/ml) = 112.6−8.2 * Age (yr), R = −0.13, p = 0.195. See also Andersson AM, Skakkebaek NE (2001) Serum inhibin B levels during male childhood and puberty. Mol Cell Endocrinol 180∶103–107. (TIF) [file pone.0020533.s002.tif]

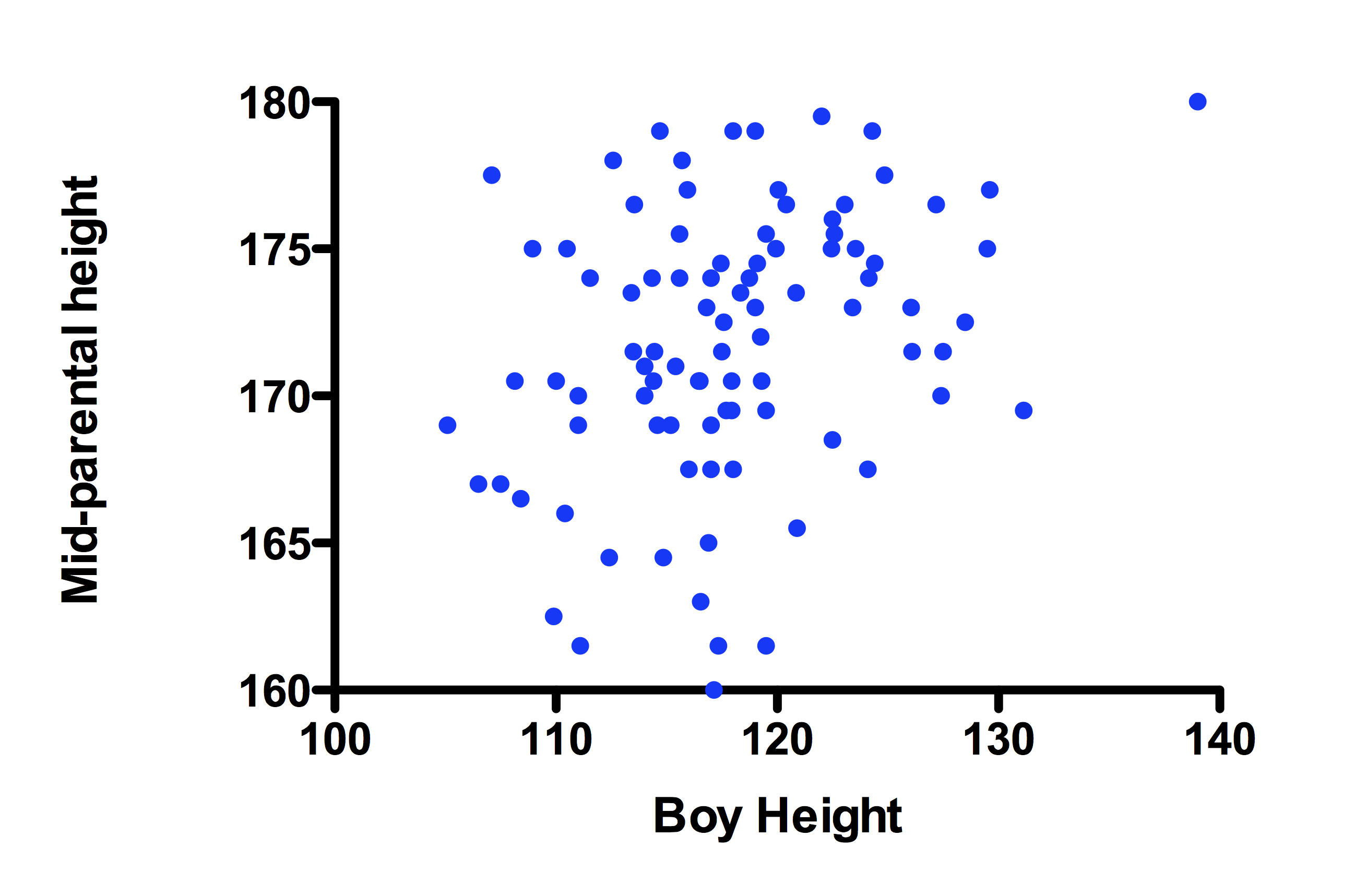

Supplement: Figure S3 — The height of 5- and 6-year-old boys is plotted against the average height of their parents. The height of the boys was significantly correlated with that of their parents, R = 0.33, p = 0.001, n = 97. (TIF) [file pone.0020533.s003.tif]

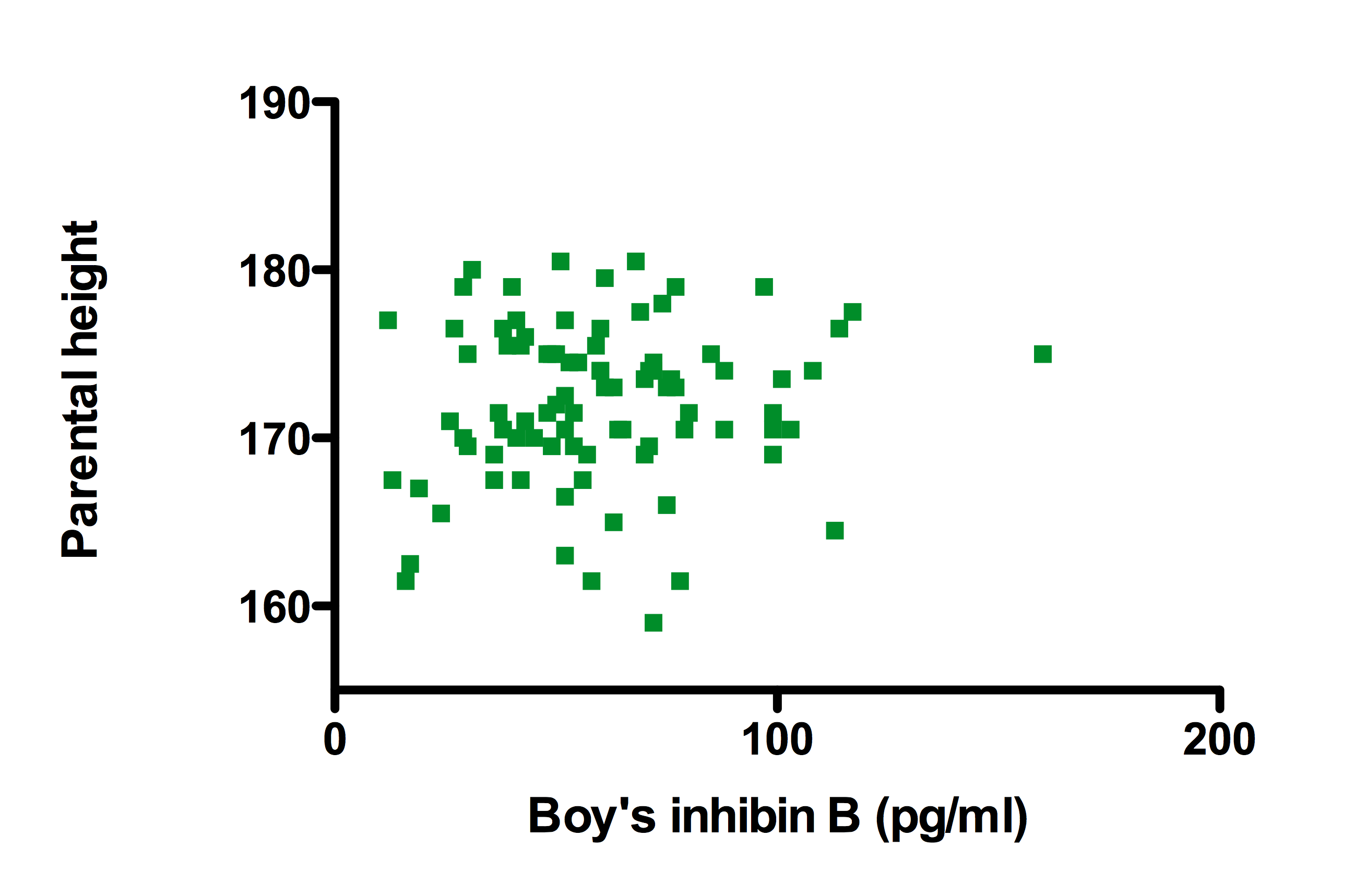

Supplement: Figure S4 — The boys' level of InhB is plotted against the height of their parents. The two variables are not significantly correlated, R = 0.03, p = 0.776. (TIF) [file pone.0020533.s004.tif]

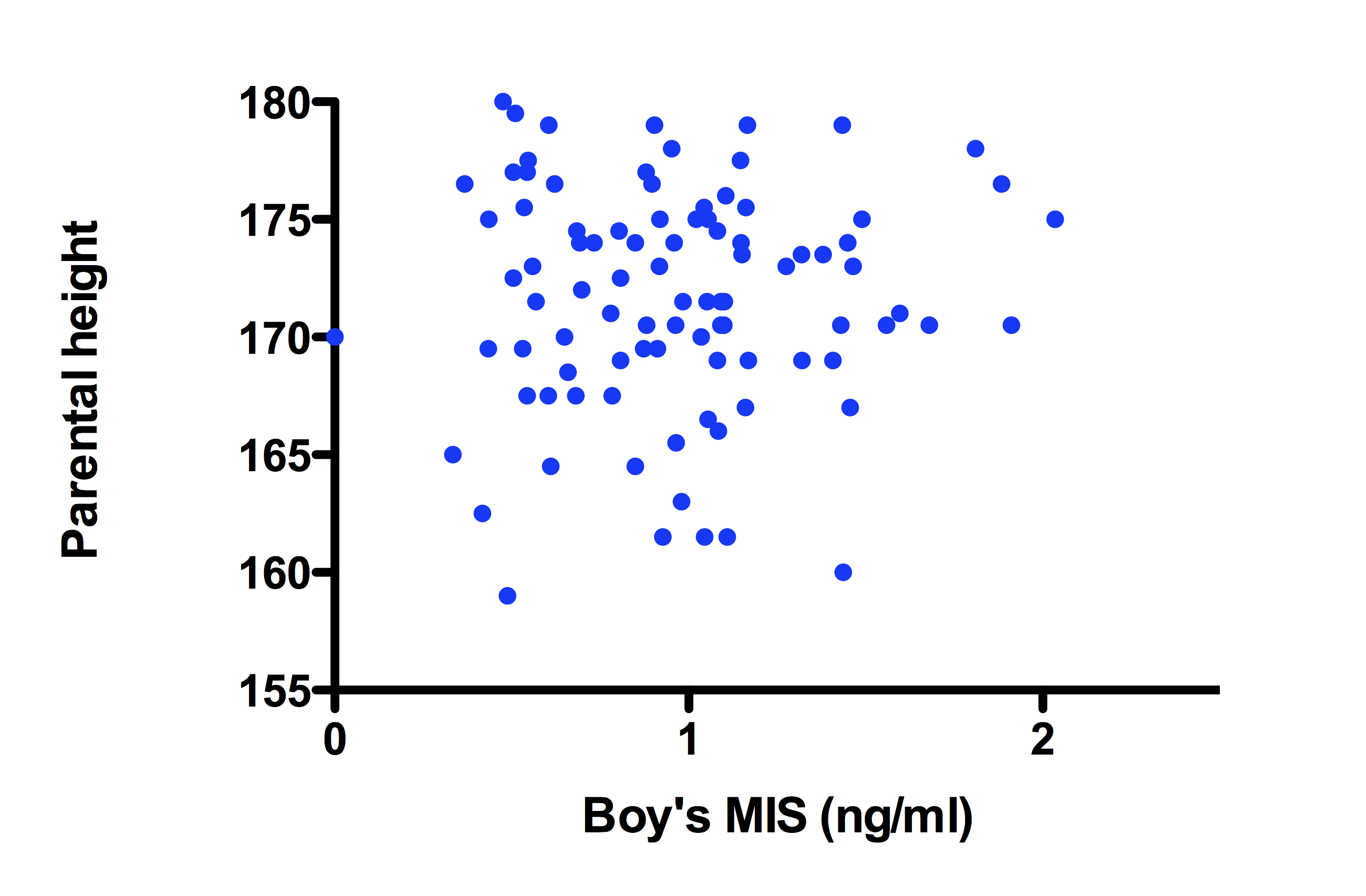

Supplement: Figure S5 — The boys' level of MIS is plotted against the height of their parents. The two variables are not significantly correlated, R = 0.01, p = 0.910. (TIF) [file pone.0020533.s005.tif]

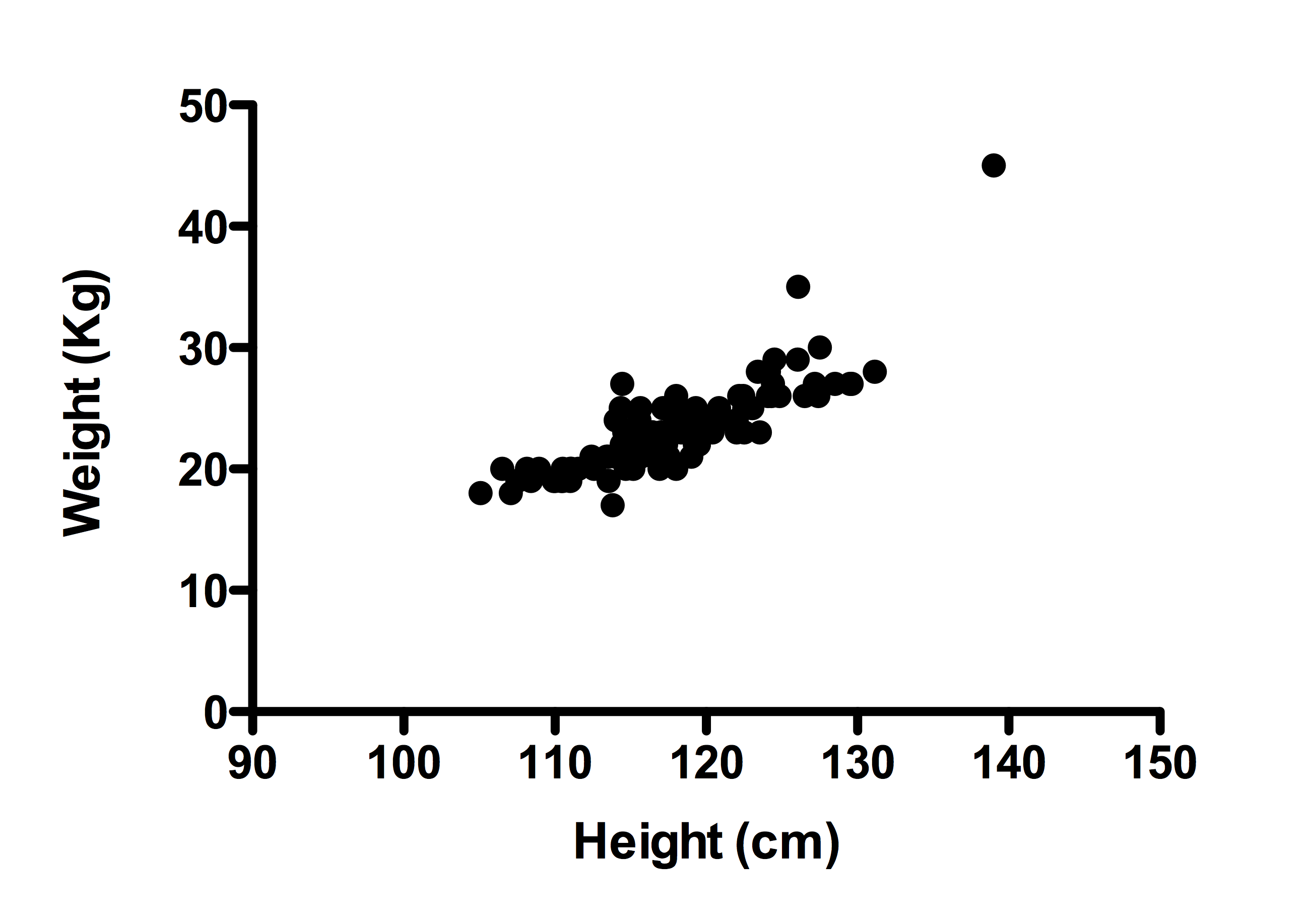

Supplement: Figure S6 — The height of the boys is plotted against their weights. The height and weight of the boys were significantly correlated, R = 0.83, p<0.0005. (TIF) [file pone.0020533.s006.tif]
