# Supplementary material for: The Stature of Boys Is Inversely Correlated to the Levels of Their Sertoli Cell Hormones: Do the Testes Restrain the Maturation of Boys?
Source: PLoS One. 2011 Jun 2;6(6):e20533. doi: 10.1371/journal.pone.0020533 (PMC3107220; doi:10.1371/journal.pone.0020533)
Supplement: Table S1 — Relationship between Sertoli cell and other hormones and weight, corrected for the boy's age. The number of boys examined is listed in Table S2. (DOC) [file pone.0020533.s007.doc]

| Correlate | Partial correlations, corrected for age | | | | | | | |
| --- | --- | --- | --- | --- | --- | --- | --- | --- |
|  | Weight | | Weight | | Benn index | | BMI | |
|  |  | | Corrected for height | | Weight/Height | | Weight/Height^2^ | |
|  | **R** | **p** | **R** | **p** | **R** | **p** | **R** | **p** |
| MIS | -0.14 |  | 0.03 |  | -0.08 |  | -0.02 |  |
| InhB | -0.13 |  | 0.05 |  | -0.06 |  | 0.06 |  |
| IGF1 | 0.41 | 0.000 | 0.03 |  | 0.42 | 0.000 | 0.29 | 0.005 |
| IGFBP3 | 0.10 |  | -0.01 |  | 0.07 |  | 0.06 |  |
| IGF1/IGFBP3 | 0.22 | 0.030 | 0.02 |  | 0.22 | 0.036 | 0.19 |  |
| iPTH | -0.18 |  | -0.23 | 0.044 | -0.16 |  | -0.22 | 0.039 |
| T3 | 0.14 |  | 0.01 |  | 0.12 |  | 0.05 |  |
| T4 | 0.06 |  | -0.02 |  | 0.02 |  | -0.02 |  |
