# Supplementary material for: The Stature of Boys Is Inversely Correlated to the Levels of Their Sertoli Cell Hormones: Do the Testes Restrain the Maturation of Boys?
Source: PLoS One. 2011 Jun 2;6(6):e20533. doi: 10.1371/journal.pone.0020533 (PMC3107220; doi:10.1371/journal.pone.0020533)
Supplement: Table S2 — Relationships between Sertoli cell hormones and other determinants of the heights of boys. Abbreviations: IGF1, insulin like growth factor 1; IGFBP3, IGF binding protein 3; iPTH, intact parathyroid hormone; T3, triiodothyronine; T4, thyroxine. (DOC) [file pone.0020533.s008.doc]

| **Correlate** | **Number** | **MIS** | | **InhB** | | **Height** | |
| --- | --- | --- | --- | --- | --- | --- | --- |
|  |  | **R** | **p** | **R** | **p** | **R** | **p** |
| **MIS** | 103 |  |  | 0.48 | 0.000 | -0.34 | 0.000 |
| **InhB** | 98 | 0.48 | 0.000 |  |  | -0.29 | 0.004 |
| **IGF1** | 91 | -0.01 |  | -0.09 |  | 0.51 | 0.000 |
| **IGFBP3** | 83 | 0.09 |  | -0.13 |  | 0.15 |  |
| **IGF1/IGFBP3** | 83 | -0.01 |  | 0.04 |  | 0.26 | 0.019 |
| **iPTH** | 84 | -0.16 |  | -0.07 |  | -0.03 |  |
| **T3** | 84 | 0.02 |  | 0.02 |  | 0.12 |  |
| **T4** | 84 | 0.07 |  | -0.09 |  | 0.14 |  |
| **Maternal height** | 98 | 0.08 |  | 0.03 |  | 0.24 | 0.018 |
| **Paternal height** | 98 | -0.05 |  | 0.00 |  | 0.20, | 0.053 |
| **Mid-parental height** | 97 | 0.01 |  | 0.03 |  | 0.33, | 0.001 |
